# A proteomic profile of synoviocyte lesions microdissected from formalin-fixed paraffin-embedded synovial tissues of rheumatoid arthritis

**DOI:** 10.1186/s12014-015-9091-8

**Published:** 2015-08-06

**Authors:** Junji Hayashi, Makoto Kihara, Harubumi Kato, Toshihide Nishimura

**Affiliations:** Niizashiki Central General Hospital, Saitama, Japan; Medical ProteoScope Co., Ltd., Tokyo, Japan; Department of Thoracic and Thyroid Surgery, Tokyo Medical University, Tokyo, Japan

**Keywords:** Rheumatoid arthritis, Osteoarthritis, Synovial tissues, Formalin-fixed paraffin embedded (FFPE), HPLC/mass spectrometry, Spectral counting, Protein–protein interaction network analysis

## Abstract

**Background:**

Rheumatoid arthritis (RA) is a systemic autoimmune disease characterized by chronic inflammation of the synovial joints. Early intervention followed by early diagnosis can result in disease remission; however, both early stage diagnosis and provision of effective treatment have been impeded by the heterogeneity of RA, which details of pathological mechanism are unclear. Regardless of numerous investigations of RA by means of genomic and proteomic approaches, proteins interplaying in RA synovial tissues that contain various types of synoviocytes, are not yet sufficiently understood. Hence we have conducted an HPLC/mass spectrometry-based exploratory proteomic analysis focusing on synoviocyte lesions laser-microdissected (LMD) from formalin-fixed paraffin-embedded (FFPE) synovial tissues (RA, *n* = 15; OA, *n* = 5), where those of Osteoarthritis (OA) were used as the control.

**Results:**

A total of 508 proteins were identified from the RA and OA groups. With the semi-quantitative comparisons, the spectral index (*SpI*), log2 protein ratio (*R*_*SC*_) based on spectral counting, and statistical G-test, 98 proteins were found to be significant (pair-wise *p* < 0.05) to the RA synovial tissues. These include stromelysin-1 (MMP3), proteins S100-A8 and S100-A9, plastin-2, galectin-3, calreticulin, cathepsin Z, HLA-A, HLA-DRB1, ferritin, neutrophil defensin 1, CD14, MMP9 etc.

**Conclusions:**

Our results confirmed the involvement of known RA biomarkers such as stromelysin-1 (MMP3) and proteins S100-A8 and S100-A9, and also that of leukocyte antigens such as HLA-DRB1. Network analyses of protein–protein interaction for those proteins significant to RA revealed a dominant participation of ribosome pathway (*p* = 5.91 × 10^−45^), and, interestingly, the associations of the p53 signaling (*p* = 2.34 × 10^−5^). An involvement of proteins including CD14, S100-A8/S100-A9 seems to suggest an activation of the NF-*k*B/MAPK signaling pathway. Our strategy of laser-microdissected FFPE-tissue proteomic analysis in Rheumatoid Arthritis thus demonstrated its technical feasibility in profiling proteins expressed in synovial tissues, which may play important roles in the RA pathogenesis.

**Electronic supplementary material:**

The online version of this article (doi:10.1186/s12014-015-9091-8) contains supplementary material, which is available to authorized users.

## Background

Rheumatoid arthritis (RA) is a systemic autoimmune disease characterized by chronic inflammation of the synovial joints, ultimately leading to joint cartilage destruction and permanent disability. It presents several systemic features as various organs are affected including skin, lungs, kidneys, blood vessels and the heart [1–4]. RA currently affects around 1 million people in Japan, [5] and approximately 1% of the population worldwide [6–8]. This inflammatory disorder is initiated by self-attack from one’s own immune system, but the details of the pathological mechanism are not clear. Recently, genomic and proteomic technologies have dramatically extended our ability to investigate the pathogenic process of RA. A series of reports has compared “fingerprint” profiles using a proteomic approach, which has found some RA-specific proteins including S100A9/A8, serum amyloid A, galectin, and ubiquitin–proteasome pathway components [9–26]. However, most of these studies were conducted with peripheral blood, synovial fluid (SF), or cultured synovial cells from patients with RA, when in fact most synoviocytes responsible for the inflammatory joint disorders in RA are found in the whole RA synovial tissue. Only a few studies have focused on the expression profile of such whole RA synovial tissue. Recent advancements in shotgun sequencing and quantitative mass spectrometry for protein analyses could make proteomics amenable to clinical biomarker discovery [27–30]. Moreover, selective collection of target cells from formalin fixed paraffin embedded (FFPE) tissues by laser microdissection (LMD) will allow access to tissues of a variety of cell types with a definite diagnosis. [31–34] Hence, we have applied this approach in order to attain a proteomic profile of RA from laser-microdissected FFPE synoviocyte lesions, which will help better understand the molecular mechanisms involved in RA.

## Results and discussion

### Proteins candidates characteristic to RA and OA

**Fig. 1:**
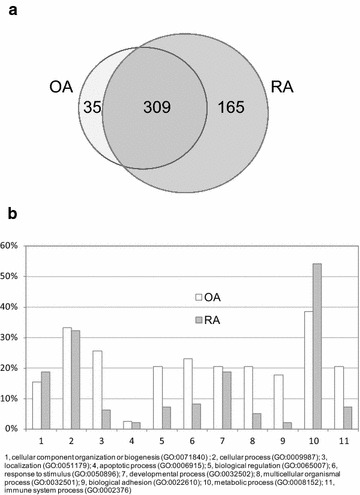
**a** The Venn diagram of 508 proteins identified from both OA and RA synovial lesions. The numbers of proteins detected with at least two peptides are indicated by *numbers*. **b** PANTHER gene ontology (GO) analysis on the biological processes of proteins preferentially expressed in OA and RA.

We have identified a total of 508 proteins from OA and RA samples, among which 165 proteins were unique to RA, 309 proteins in common, and only 35 unique to OA, as shown in Fig. 1a. These proteins were subjected to Protein ANalysis THrough Evolutionary Relationships (PANTHER) Classification System version 9.0, [35] highlighting their biological processes. As Fig. 1b shows, large differences were found at the following biological processes of proteins characteristically expressed in the RA vs. OA pair: 3, localization (GO:0051179); 5, biological regulation (GO:0065007); 6, response to stimulus (GO:0050896); 8, multicellular organismal process (GO:0032501); 9, biological adhesion (GO:0022610); 11, immune system process (GO:0002376). Differential protein expression analysis has been performed by using the spectral index (*SpI*), [36] the fold change of a expressed protein in the base 2 logarithmic scale (*R*_*SC*_) [37] which are based on spectral counting. G test was used for evaluating differential protein expression in pair-wise, RA vs. OA [38].

A protein characteristic to either group was defined to satisfy *p* < 0.05 in pairwise G-test, and *R*_*SC*_ > 1 or < −1, under which criterions 98 proteins were characteristic to RA and 71 proteins to OA among 508 identified proteins. The list of total 169 proteins is given in Additional file 1: Table S4, in which contained numerous RA biomarkers known previously, for example, stromelysin-1 (MMP3) and proteins S100-A8 and S100-A9, and so on. Table 1 lists the 31 proteins expressed in RA and OA with the significance of *p* values <0.0001 in G test, and under *R*_*SC*_ values >2 or <−2.

**Table 1:** Representative 31 proteins with *p* values <0.0001 in G test, and *R*_*SC*_ values >2 or <−2, which means the fold change of a protein higher than 4 or lower than 0.25, in pairwise comparison between RA and OA

Protein entry name^a^	Gene ID	Description	Length of amino acid	Number of samples in which a protein was identified		Spectral counts (*SpC*s)		*p* value in G-test	Spectral Index (*SpI*)	*R*_*SC*_
				OA (n = 5)	RA (n = 15)	OA-group	RA-group			
1A33	HLA-A	HLA class I histocompatibility antigen, A-33 alpha chain	365	1	12	2	84	7.667E−08	0.777	2.904
TXND5	TXNDC5	Thioredoxin domain-containing protein 5	432	2	12	4	87	1.929E−06	0.747	2.261
DEF1	DEFA1	Neutrophil defensin 1	94	0	11	0	78	4.879E−10	0.733	4.177
RL7A	RPL7A	60S ribosomal protein L7a	266	0	10	0	37	2.032E−05	0.667	3.125
TPM3	TPM3	Tropomyosin alpha-3 chain	284	1	10	5	102	4.128E−07	0.626	2.237
S10A8	S100A8	Protein S100-A8	93	0	9	0	85	8.08E−11	0.600	4.299
VINC	VCL	Vinculin	1,134	1	9	3	70	1.372E−05	0.567	2.257
S10A9	S100A9	Protein S100-A9	114	0	8	0	109	1.732E−13	0.533	4.654
EZRI	EZR	Ezrin	586	0	8	0	69	4.951E−09	0.533	4.003
FRIH	FTH1	Ferritin heavy chain	183	0	8	0	40	9.239E−06	0.533	3.234
K2C6A	KRT6A	Keratin, type II cytoskeletal 6A	564	0	7	0	156	1.084E−18	0.467	5.168
PERP1	PACAP	Plasma cell-induced resident endoplasmic reticulum protein	189	0	6	0	43	4.21E−06	0.400	3.335
PERM	MPO	Myeloperoxidase	745	0	5	0	65	1.39E−08	0.333	3.918
1C12	HLA-C	HLA class I histocompatibility antigen, Cw-12 alpha chain	366	0	5	0	46	1.922E−06	0.333	3.430
K2C5	KRT5	Keratin, type II cytoskeletal 5	590	0	4	0	93	1.039E−11	0.267	4.427
TRFL	LTF	Lactotransferrin	710	0	3	0	98	2.886E−12	0.200	4.502
K1C14	KRT14	Keratin, type I cytoskeletal 14	472	0	3	0	81	2.257E−10	0.200	4.231
ACTN1	ACTN1	Alpha-actinin-1	892	0	3	0	47	1.481E−06	0.200	3.460
K2C6C	KRT6C	Keratin, type II cytoskeletal 6C	564	0	1	0	64	1.799E−08	0.067	3.896
POSTN	POSTN	Periostin	836	2	5	17	13	5.517E−05	−0.082	−2.170
CBPQ	CPQ	Plasma glutamate carboxypeptidase	472	1	0	6	0	6.486E−05	−0.200	−4.349
AEBP1	AEBP1	Adipocyte enhancer-binding protein 1	1,158	1	0	6	0	6.486E−05	−0.200	−4.349
COFA1	COL15A1	Collagen alpha-1 (XV) chain	1,388	1	0	7	0	1.354E−05	−0.200	−4.535
MYH11	MYH11	Myosin-11	1,972	1	0	10	0	1.273E−07	−0.200	−4.983
HBG2	HBG2	Hemoglobin subunit gamma-2	147	1	0	25	0	1.278E−17	−0.200	−6.207
ASPN	ASPN	Asporin	380	2	2	24	12	1.693E−08	−0.222	−2.744
HBD	HBD	Hemoglobin subunit delta	147	4	5	245	216	9.342E−48	−0.269	−2.012
CO4A	C4A	Complement C4-A	1,744	2	1	13	4	3.329E−06	−0.290	−3.254
COCA1	COL12A1	Collagen alpha-1 (XII) chain	3,063	2	1	17	5	6.862E−08	−0.294	−3.359
COEA1	COL14A1	Collagen alpha-1 (XIV) chain	1,796	4	6	167	108	4.41E−43	−0.329	−2.449
FMOD	FMOD	Fibromodulin	376	2	0	8	0	2.845E−06	−0.400	−4.700

^a^Proteins are listed in descending order of *SpI*-value, and “_HUMAN” are removed from UniProtKG entry names.

A co-expression of both fibromodulin (FMOD) and biglycan (PGS1) observed in OA-group seems consistent with the recent study that those ECM proteins were suggested by using the genetic mouse model to be essential in regulating chondrogenesis and extracellular matrix turnover in temporomandibular joint (TMJ) osteoarthritis [39]. It was also reported that asporin, (also known as periodontal ligament-associated protein 1 (PLAP1), a member of the family of small leucine-rich proteoglycan (SLRP) family), is expressed within the cartilage extracellular matrix (ECM) and have a genetic association with osteoarthritis [40].

The proteins, S100-A8 and -A9 (also known as myeloid-related protein 8 and 14, and calgranulin A and B) identified from RA synovial tissues were previously reported as biomarker candidates in RA sera, plasmas and synovial fluids [41]. Other known RA biomarker candidates were also detected, including thioredoxin domain-containing protein 5 (TXND5) [42] and thioredoxin-dependent peroxide reductase, mitochondrial (PRDX3).

### Network analysis of candidate proteins

**Fig. 2:**
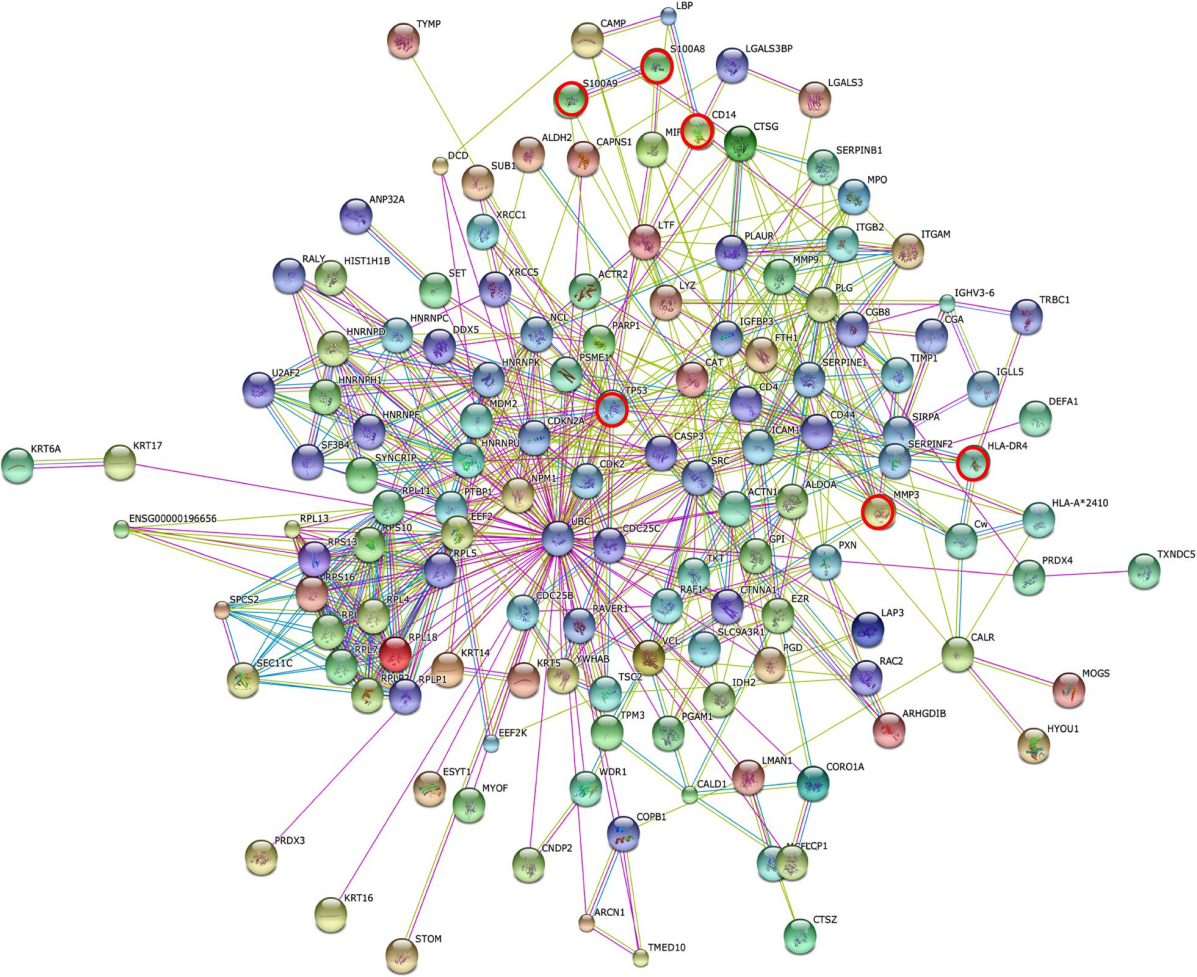
STRING protein–protein interaction networks of 98 differentially expressed proteins in RA synoviocyte lesions. This interaction map shown in evidence view was generated using default setting in network depth of 50 interactions under medium confidence (0.4) and the criteria for linkage only including experiments, databases, and textmining. Node proteins of potentially importance in RA were indicated by *red circles*.

Network analysis of significant proteins is helpful in understanding how these proteins interplay with other key proteins and pathways. This study utilized significant proteins (*n* = 98) relevant in RA to develop a predictive network model, which has the potential to be used for further biological investigation. This was done using Search Tool for the Retrieval of Interacting Genes/Proteins (STRING) database, [43, 44] in which data were obtained from biological functions of local networks surrounding the protein candidates. The STRING network of proteins differentially expressed in RA is shown in Fig. 2, where node proteins of potentially importance in RA are indicated by red circles. STRING network enrichment analyses suggested a preferable association of RA with hematopoietic system disease (DOID 74: *p* = 3.53 × 10^−10^) and immune system disease (DOID 2914: *p* = 5.28 × 10^−9^). Enrichment analyses on the KEGG pathways indicated that RA was dominantly associated with ribosome (has03010: *p* = 5.91 × 10^−45^). Interestingly, such would indicate that RA may involve protein networks that interplay with both p53 signaling (has04115: *p* = 2.34 × 10^−5^) and leukocyte transendothelial migration (has04670: *p* = 5.75 × 10^−4^).

Recent therapeutic interventions for RA have been targeting cytokines, such as TNF-a, IL-1 and IL-17, a regulating matrix degradation. Numerous studies using anti-TNF agents have shown to slow or prevent the progression of bone and cartilage damage in RA, which could be attributed to the suppression of osteoclasts in joint lesions. Besides that, it have been reported that the pathogenesis of bone erosions in RA relates to the osteoclast-mediated bone resorption that is regulated by RANKL, the RANK (receptor activator of nuclear factor (NF)-*k*B) ligand [45]. RANKL is expressed by a variety of cell types involved in RA, including T-cells and synoviocytes. NF-*k*B is activated in the synovium of patients with RA [12, 46] and regulates genes including TNF-a, IL-6, IL-8, inducible nitric oxidase synthase (iNOS) and cyclooxygenase-2 (COX-2), all of which contribute to inflammation. It should be noted that the mitogen-activated protein (MAP) kinases are also regulators of cytokine and metalloproteinase production [47, 48]. AKT2, IL6, MAPK1 and TP53 are all associated with the drugs used in RA treatment. It is known that methotrexate (MTX) causes single- and double-strand DNA breaks, which are associated with TP53 [49–51]. It is also known that the p53 pathway is affected by bucillamine (Buc), which is mainly used for pain-reduction purposes as part of RA treatment in Japan [52].

Several proteins identified as being specific to the RA-group are those related to human leukocyte antigens, such as HLA class I histocompatibility antigens, Cw-12 (HLA-C 1C12) and A-33 (HLA-A), and HLA class II histocompatibility antigen, DRB1-4 βα (HLA-DRB1). It has been considered that genetic similarities between RA patients and specific human leukocyte antigen (HLA)-DR genes, [12, 53], which reside in the major histocompatibility complex (MHC) and participate in antigen presentation, are associated with RA. The protein 2B14 is of the DRB1-4 β chain corresponding to the third hypervariable region, in which the susceptibility epitope may also influence the severity of the disease, and by which the strongest genetic link is suggested between the MHC and RA [54].

S100-A8 and S100-A9 (calgranulins, MRP8 and MRP14) are prominently released by activated macrophages. Inflammatory mediators such as IL-1, tumor necrosis factor (TNF) α or interferon (IFN) γ stimulate macrophages to up-regulate and secrete S100A8/S100A9, which induces proinflammatory responses in leucocytes and endothelial cells [55, 56]. One of the RA-related proteins identified in this study includes CD14 (monocyte differentiation antigen), which is involved in Toll-like receptor signaling [57]. It has been reported that Toll-like receptor (TLR) 4 is the dominant receptor for S100A8 signaling, and that stimulation by S100A8/S100A9 leads to nuclear factor (NF)*k*B and MAP kinase (MAPK) signalling [58, 59]. S100A8 and S100A9 and the heterodimer accumulate in inflammatory fluids, suggesting that those are involved in the pathogenesis of rheumatoid arthritis [60].

## Conclusions

We have employed in this study an exploratory proteomic analysis of laser-microdissected FFPE-tissues to elucidate protein expression profiles at synoviocyte lesions obtained from RA and OA patients, in which the OA samples served as the control. To the best of our knowledge, this is the first proteomic study that has used FFPE synovial tissues of both RA and OA. Among a total of 508 proteins identified we have elucidated 98 and 71 significant proteins (*p* < 0.05 and *R*_*SC*_ > 1 or −1) expressed in RA and OA, respectively. Molecular mechanisms leading to RA development involve quite a complex and diverse protein network interactions and thus is not yet completely understood. Identification, quantification and functional characterization of proteins are essential in further understanding RA pathogenesis. Our results confirmed the involvement of known RA biomarkers such as stromelysin-1 (MMP3) and proteins S100-A8 and S100-A9, and also that of leukocyte antigens such as HLA-DRB1. The STRING protein–protein network analysis on RA indicated the dominant participation of ribosome pathway, and, interestingly, highlighted the associations of both the p53 signaling and NF*k*B/MAPK signaling pathways. We have successfully identified several proteins expressed in RA synovial tissues, which may play important roles in RA pathogenesis. These results will help provide additional information about the molecular mechanisms of RA and improve diagnostic strategies in the future. Thus laser-microdissected FFPE-tissue proteomic analysis has its position as a technically feasible method in this area and further research including exploratory and validation studies in individual patients in larger populations across multiple locations should be carried out in the future.

## Methods

### Ethics approval

The study protocol conformed to the principles of the Declaration of Helsinki. All patients were provided with informed consent and the study protocol was approved by both the Niizashiki Central General Hospital ethics committee and Medical ProteoScope Co. Ltd. Ethical committee.

### Patients’ characteristics

Synovial tissue samples were obtained from patients with RA (*n* = 15) and OA (*n* = 5) undergoing a variety of orthopedic surgery (wrist joint, elbow joint, hip joint, knee joint) at the Niiza Shiki Central general hospital. All patients fulfilled the American College of Rheumatology criteria for the diagnoses of RA and OA [61–63]. Table 2 summarizes patients’ characteristics and clinical information.

**Table 2:** Patients’ characteristics and clinical information

	RA (*n* = 15)	OA (*n* = 5)
Age (years)	62.9 ± 10.3	66.2 ± 9.7
Gender, n (% female)	11 (73.3)	5 (100.0)
Disease duration (years)	11.7 ± 9.0	5.7 ± 5.6
Stage (I/II/III/IV)	1/1/11/2	
Class (I/II/III/IV)	1/5/8/1	
DAS28-CRP	3.5 ± 1.1	
CRP(mg/dL)	2.4 ± 2.2	
MMP-3 (ng/mL)	430.8 ± 531.5	
MTX use, n (%)	9 (60.0)	
MTX dose (mg/week)	4.1 ± 3.6	
Oral steroid use, n (%)	7 (46.7)	
Oral steroid dose (mg/day)	1.4 ± 1.7	
Biologics use, n (%)	3 (20.0)	
Sampling site		
Wrist joint, n	3	0
Elbow joint, n	1	0
Hip joint, n	1	4
Knee joint, n	10	1

The values are mean ± SD unless otherwise indicated.

*DAS* disease activity score, *CRP* C-reactive protein, *MMP-3* matrix metalloproteinase 3, *MTX* methotrexate.

### FFPE tissue sample preparation

The synovial samples were dissected from connective tissues and immediately stored at −80°C until use. Synovial tissues were then surgically removed and fixed with a buffered formalin solution containing 10–15% methanol and were finally embedded by a conventional method. Paraffin blocks were cut into 4-μm sections for diagnosis and 10-μm sections for proteomics. The 10-μm sections were stained only with haematoxylin, and diagnosis made using the 4-μm sections stained with haematoxylin-eosin (HE) according to the WHO classification.

### Laser capture and protein solubilization

**Fig. 3:**
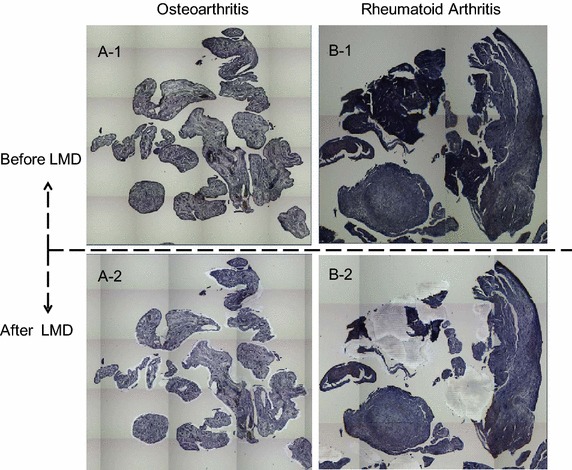
Examples of the laser microdissections (LMDs) of targeted lesions from **a** OA and **b** RA synovial tissues (1, before; 2, after) on the DIRECTOR^®^ slides. The DIRECTOR^®^ slide is similar to a standard glass (uncharged) microscope slide, but has an energy transfer coating on one side of the slide. Tissue sections are mounted on top of the energy transfer coating, and when the slide is turned over, the tissue faces down under the microdissection system. Targeting cells or tissue areas of interest is carried out on computer display. The laser energy is converted to kinetic energy upon striking the coating, vaporizing it and instantly propelling selected tissue features into the collection tube.

Targeted synoviocyte lesions were identified on serial sections of synovial tissues stained with hematoxylin and eosin (HE). For proteomic analysis, a 10-μm thick section prepared from the same tissue block was attached onto DIRECTOR^®^slides (OncoPlexDx, Rockville, MD, USA), de-paraffinized twice with xylene for 5 min, rehydrated with graded ethanol solutions and distilled water, and stained by hematoxylin. Those slides were air-dried and subjected to laser microdissection with a Leica LMD6000 (Leica Micro-systems GmbH, Ernst-Leitz-Strasse, Wetzlar, Germany). The DIRECTOR^®^ slide is similar to a standard glass (uncharged) microscope slide, but has an energy transfer coating on one side of the slide. Tissue sections are mounted on top of the energy transfer coating, and when the slide is turned over, the tissue faces down under the microdissection system. Targeting cells or tissue areas of interest are carried out on computer display. The laser energy is converted to kinetic energy upon striking the coating, vaporizing it and instantly propelling selected tissue features into the collection tube. At least 30,000 cells (ca. 8.0 mm^2^) were collected directly into a 1.5-mL low-binding plastic tube. Proteins were extracted and digested with trypsin using Liquid Tissue^®^ MS Protein Prep kits (OncoPlexDx, Rockville, MD, USA) according to the manufacturer’s protocol. Targeted lesions were laser-microdissected from FFPE synovial tissues as exemplified in Fig. 3.

### Liquid chromatography-tandem mass spectrometry

We adopted a label-free semi-quantitation using spectral counting by liquid chromatography (LC)-tandem mass spectrometry (MS/MS) to a global proteomic analysis. The digested samples were analyzed in triplicates and orders randomized by LC–MS/MS using reversed-phase liquid chromatography (Paradigm MS4; Michrom Bioresources, USA) (RP-LC) interfaced with a LTQ-Orbitrap XL hybrid mass spectrometer (Thermo Fisher Scientific, Bremen, Germany) via a closed *nano*-electrospray device (ADVANCE Spray Source; AMR Inc. Japan) as described in details previously [64]. Briefly, the RP-LC system consisted of a peptide Cap-Trap cartridge (0.3 × 5.0 mm) and a capillary separation column (an L-column Micro of 0.1 × 150 mm packed with reverse phase L-C18 gels of 3 μm in diameter and 12-nm pore size, (CERI, Tokyo, Japan)). An autosampler (HTC-PAL, CTC Analytics, Switzerland) loaded an aliquot of samples onto the trap, which was then washed with solvent A (98% distilled water with 2% acetonitrile and 0.1% formic acid) for concentrating peptides on the trap and desalting. The trap was subsequently connected in series to the separation column, and the whole columns were developed for 100 min with a linear acetonitrile concentration gradient made from 5 to 35% solvent B (10% distilled water and 90% acetonitrile containing 0.1% formic acid) at the flow-rate of 300 nL/min. A 5 μL (corresponding to 1/10 of total sample amount) was used for each LC–MS analysis.

An LTQ was operated in the data-dependent MS/MS mode to automatically acquire up to three successive MS/MS scans in the centroid mode. The three most intense precursor ions for these MS/MS scans could be selected from a high-resolution MS spectrum (a survey scan) that an Orbitrap previously acquired during a predefined short time window in the profile mode at the resolution of 30,000 and the lock mass of *m*/*z* 536.1,654 in the *m*/*z* range of 350–1,500. The sets of acquired high-resolution MS and MS/MS spectra for peptides were converted to single data files and they were merged into Mascot generic format files for database searching.

### Database search

Mascot software (version 2.2.06, Matrix Science, London, UK) was used for database search against Homo sapiens entries in the UniProtKB/Swiss-Prot database (release 2012_02, 20413 entries). Peptide mass tolerance was 5 ppm, fragment mass tolerance 0.5 Da, and up to two missed cleavages were allowed for errors in trypsin specificity. Carbamidomethylation of cysteines was taken as fixed modifications, and methionine oxidation and formylation of lysine, arginine and N-terminal amino acids as variable modifications. A *p* values of < 0.05 was considered significant, lists of identified proteins were made under the criterions, peptide probability >95%, protein probability >99% and 2 minimum unique peptides, and then were merged into a master file where the primary accession numbers and entry names from UniProtKB were used. The false positive rates for protein identification were estimated using a decoy database created by reversing the protein sequences in the original database; the estimated false positive rate of peptide matches was 0.2% under protein score threshold conditions (*p* < 0.001).

### Semi-quantitative group-comparison with spectral counting

Mascot search results were processed through Scaffold software (version 3.3.3, Proteome Software, Portland, OR, USA) to semi-quantitatively analyze differential expression levels of proteins by the spectral counting as described [32]. The number of peptide MS/MS spectra with high confidence (Mascot ion score, *p* < 0.005) was used for calculating spectral counts. Differential protein expression analysis was performed by the spectral index, *SpI*, which takes into account non-normal distribution and limited replicates and/or sample sizes [36]. *SpI* takes a value between −1 to 1, and a protein of *SpI* > 0.4 or <−0.4 are considered to be significant. *R*_*SC*_ > 1 or <−1 corresponds to their fold changes >2 or <0.5. G test was used for evaluating differential protein expression in pair-wise cancer groups [38]. Although G test does not require replicates, spectral counts for each protein from triplicates were pooled and used for G-statistic calculation using a two-way contingency table arranged in two rows for a target protein and any other proteins, and two columns for cancer groups on an Excel macro. Statistical significance of *p* < 0.05 was used. The Yates correction for continuity was applied to the 2 × 2 tables. The spectral counts were calculated for identified proteins, and those from triplicate experiments were pooled, thereby improving the performance of G-test and decreasing false positive rates significantly [38].

The correction has made handling of data containing small spectral counts, including zero, possible. Statisticians, however, showed that the results of G-test using a contingency table containing small counts are not so convincing due to the assumption that the G statistic asymptotically obey a χ^2^ distribution with one degree of freedom. To validate the G-test results, we calculated exact *p* values for the significant proteins without making any assumptions of statistical distribution, based on the permutational distribution of the test statistic, i.e., Fisher’s exact test and Mann–Whitney U test for the contingency tables using a R package.

### Network analysis of protein–protein interactions

Network analysis of protein–protein interactions was carried out by using STRING version 9.1, [43] in which nodes are proteins and edges are the predicted functional associations based on primary databases comprising of KEGG and GO, and primary literature. STRING predicts these interactions based on neighbourhood, gene fusion products, homology and similarity of coexpression patterning. Network interaction scores for each node are expressed as a joint probability derived from curated databases of experimental information, text mining and computationally predicted by genetic proximity [44]. In this study, STRING networks were calculated with the default settings—medium confidence score: 0.400, network depth: 0 and up to 50 interactions.

## Additional file

Additional file 1. In the Supplemental Material Section presented are the total laser-microdissection (LMD) areas (Supplemental Table 1), the average retention time (RT) and CV of each of the 11 representative peptides (Supplemental Table 2), total and average spectral counts per run and the corresponding CV in triplicate runs (Supplemental Table 3), the 169 proteins expressed with *p* values < 0.05 in G-statistics and *R*_*SC*_ values > 1 or <−1 (Supplemental Table 4), the example of TIC chromatographic profiles (Supplemental Figure 1) and the fold changes of three of the representative proteins (S100A8, RS9 and PERP1) in log2 comparing the peak areas extracted from LC-MS raw data with the spectral counts (Supplemental Figure 2).

## Abbreviations

RA: rheumatoid arthritis
OA: osteoarthritis
DAS: disease activity score in rheumatoid arthritis
FFPE: formalin-fixed paraffin embedded
LMD: laser-microdissection
HPLC: high pressure liquid chromatography
MS/MS: tandem mass spectrometry
GO: gene ontology
PANTHER: protein analysis through evolutionary relationships classification system
DOID: disease ontology ID
hsa: homo sapiens
STRING: search tool for the retrieval of interacting genes/proteins database
