# Supplementary material for: A proteomic profile of synoviocyte lesions microdissected from formalin-fixed paraffin-embedded synovial tissues of rheumatoid arthritis
Source: Clin Proteomics. 2015 Aug 6;12(1):20. doi: 10.1186/s12014-015-9091-8 (PMC4527102; doi:10.1186/s12014-015-9091-8)
Supplement: Additional file 1. — In the Supplemental Material Section presented are the total laser-microdissection (LMD) areas (Supplemental Table 1), the average retention time (RT) and CV of each of the 11 representative peptides (Supplemental Table 2), total and average spectral counts per run and the corresponding CV in triplicate runs (Supplemental Table 3), the 169 proteins expressed with p values < 0.05 in G-statistics and R SC values > 1 or <−1 (Supplemental Table 4), the example of TIC chromatographic profiles (Supplemental Figure 1) and the fold changes of three of the representative proteins (S100A8, RS9 and PERP1) in log2 comparing the peak areas extracted from LC-MS raw data with the spectral counts (Supplemental Figure 2). [file 12014_2015_9091_MOESM1_ESM.doc]

**SUPPLEMENTAL MATERIALS:**

Supplemental Table 1. Specimen IDs and their recorded total area（μm^2^) from laser-microdissection (LMD).

| Specimen ID | Recorded Total area  （μm^2^) |
| --- | --- |
| RA01 | 8,153,724 |
| RA02 | 8,160,829 |
| RA03 | 8,162,778 |
| RA04 | 8,151,575 |
| RA05 | 8,153,779 |
| RA06 | 8,158,138 |
| RA07 | 8,152,065 |
| RA08 | 8,159,837 |
| RA09 | 8,140,127 |
| RA10 | 8,166,674 |
| RA11 | 8,161,643 |
| RA12 | 8,153,564 |
| RA13 | 8,146,387 |
| RA14 | 8,158,194 |
| RA15 | 8,150,565 |
| OA01 | 8,143,460 |
| OA02 | 8,148,479 |
| OA03 | 8,124,353 |
| OA04 | 8,148,498 |
| OA05 | 8,166,773 |

Supplemental Table 2. The average retention time (RT) and CV of each of the 11 representative peptides measured throughout ~~all~~ the 20 samples. The triplicate LC/MS runs ranged from 8.793 min to 92.812 min.

| No. | Protein name | Peptide Sequence | Average RT | CV |
| --- | --- | --- | --- | --- |
| 1 | CALR_HUMAN | R.LKEEEEDKK.R | 8.793 | 0.014 |
| 2 | ACTG_HUMAN | K.DSYVGDEAQSK.R | 19.614 | 0.086 |
| 3 | TCTE1_HUMAN | R.SKVDDDKAR.I + 2 Formyl (K) | 24.370 | 0.062 |
| 4 | EF1A1_HUMAN | K.STTTGHLIYK.C | 27.042 | 0.013 |
| 5 | ANXA2_HUMAN | K.TPAQYDASELK.A | 34.156 | 0.013 |
| 6 | PLEC_HUMAN | K.LTVEEAVR.M | 38.067 | 0.008 |
| 7 | TBB5_HUMAN | K.EVDEQMLNVQNK.N | 43.307 | 0.007 |
| 8 | H4_HUMAN | R.ISGLIYEETR.G | 52.872 | 0.024 |
| 9 | MYH9_HUMAN | R.RGDLPFVVPR.R | 58.023 | 0.005 |
| 10 | LAC6_HUMAN | K.YAASSYLSLTPEQWK.S | 75.864 | 0.010 |
| 11 | BGH3_HUMAN | R.LTLLAPLNSVFK.D | 92.812 | 0.010 |

Supplemental Table 3. Total and Average spectral counts per run, and the corresponding CV of triplicate runs.

| Group | Sample Number_Run Number | Total Spectral Counts per Run | Average Total Spectral Counts | CV |
| --- | --- | --- | --- | --- |
| OA | OA01_1 | 940 | 935.0 | 0.100 |
|  | OA01_2 | 1026 |  |  |
|  | OA01_3 | 839 |  |  |
|  | OA02_1 | 576 | 575.3 | 0.002 |
|  | OA02_2 | 574 |  |  |
|  | OA02_3 | 576 |  |  |
|  | OA03_1 | 1167 | 1169.0 | 0.028 |
|  | OA03_2 | 1137 |  |  |
|  | OA03_3 | 1203 |  |  |
|  | OA04_1 | 1152 | 1175.3 | 0.025 |
|  | OA04_2 | 1209 |  |  |
|  | OA04_3 | 1165 |  |  |
|  | OA05_1 | 798 | 764.3 | 0.041 |
|  | OA05_2 | 758 |  |  |
|  | OA05_3 | 737 |  |  |
| RA | RA01_1 | 1121 | 1073.3 | 0.069 |
|  | RA01_2 | 1111 |  |  |
|  | RA01_3 | 988 |  |  |
|  | RA02_1 | 888 | 870.3 | 0.018 |
|  | RA02_2 | 860 |  |  |
|  | RA02_3 | 863 |  |  |
|  | RA03_1 | 898 | 870.3 | 0.082 |
|  | RA03_2 | 924 |  |  |
|  | RA03_3 | 789 |  |  |
|  | RA04_1 | 1069 | 1058.0 | 0.072 |
|  | RA04_2 | 1128 |  |  |
|  | RA04_3 | 977 |  |  |
|  | RA05_1 | 1036 | 1023.7 | 0.040 |
|  | RA05_2 | 1057 |  |  |
|  | RA05_3 | 978 |  |  |
|  | RA06_1 | 763 | 740.7 | 0.057 |
|  | RA06_2 | 767 |  |  |
|  | RA06_3 | 692 |  |  |
|  | RA07_1 | 1161 | 1159.0 | 0.016 |
|  | RA07_2 | 1176 |  |  |
|  | RA07_3 | 1140 |  |  |
|  | RA08_1 | 1263 | 1240.3 | 0.020 |
|  | RA08_2 | 1245 |  |  |
|  | RA08_3 | 1213 |  |  |
|  | RA09_1 | 1181 | 1181.3 | 0.037 |
|  | RA09_2 | 1225 |  |  |
|  | RA09_3 | 1138 |  |  |
|  | RA10_1 | 1169 | 1224.7 | 0.040 |
|  | RA10_2 | 1261 |  |  |
|  | RA10_3 | 1244 |  |  |
|  | RA11_1 | 1087 | 1127.3 | 0.039 |
|  | RA11_2 | 1175 |  |  |
|  | RA11_3 | 1120 |  |  |
|  | RA12_1 | 1266 | 1182.3 | 0.079 |
|  | RA12_2 | 1200 |  |  |
|  | RA12_3 | 1081 |  |  |
|  | RA13_1 | 1153 | 1076.7 | 0.101 |
|  | RA13_2 | 1125 |  |  |
|  | RA13_3 | 952 |  |  |
|  | RA14_1 | 1210 | 1207.0 | 0.010 |
|  | RA14_2 | 1217 |  |  |
|  | RA14_3 | 1194 |  |  |
|  | RA15_1 | 1164 | 1183.3 | 0.017 |
|  | RA15_2 | 1204 |  |  |
|  | RA15_3 | 1182 |  |  |

Supplemental Table 4. 169 proteins with *p*-values < 0.05 in G-statistics and *R_SC_* -values > 1 or <-1 (i.e. fold change of a protein that is higher than 2 or lower than 0.5) in pairwise comparison between RA and OA.

| Protein Entry Name | Gene ID | Description | Length of amino acid | Number of samples in which a protein was identified | |  | Spectral counts (*SpC*s) | | *p*-value in G-test | Spectral Index (*SpI*) | *R_SC_* |
| --- | --- | --- | --- | --- | --- | --- | --- | --- | --- | --- | --- |
|  |  |  |  | OA (n=5) | RA (n=15) |  | OA-group | RA-group |  |  |  |
| HNRPD | HNRNPD | Heterogeneous nuclear ribonucleoprotein D0 | 355 | 1 | 13 |  | 2 | 51 | 1.519E-04 | 0.826 | 2.196 |
| PLSL | LCP1 | Plastin-2 | 627 | 3 | 14 |  | 15 | 193 | 5.200E-09 | 0.823 | 1.772 |
| CALR | CALR | Calreticulin | 417 | 2 | 14 |  | 13 | 140 | 6.589E-06 | 0.820 | 1.500 |
| CATZ | CTSZ | Cathepsin Z | 303 | 1 | 13 |  | 4 | 71 | 5.558E-05 | 0.810 | 1.972 |
| ALDOA | ALDOA | Fructose-bisphosphate aldolase A | 364 | 2 | 14 |  | 8 | 66 | 1.125E-02 | 0.789 | 1.051 |
| TYPH | TYMP | Thymidine phosphorylase | 482 | 3 | 14 |  | 18 | 173 | 3.300E-06 | 0.789 | 1.370 |
| 1A33 | HLA-A | HLA class I histocompatibility antigen, A-33 alpha chain | 365 | 1 | 12 |  | 2 | 84 | 7.667E-08 | 0.777 | 2.904 |
| RLA2 | RPLP2 | 60S acidic ribosomal protein P2 | 115 | 1 | 13 |  | 6 | 59 | 6.021E-03 | 0.768 | 1.244 |
| TXND5 | TXNDC5 | Thioredoxin domain-containing protein 5 | 432 | 2 | 12 |  | 4 | 87 | 1.929E-06 | 0.747 | 2.261 |
| DEF1 | DEFA1 | Neutrophil defensin 1 | 94 | 0 | 11 |  | 0 | 78 | 4.879E-10 | 0.733 | 4.177 |
| G6PI | GPI | Glucose-6-phosphate isomerase | 558 | 2 | 13 |  | 12 | 94 | 3.860E-03 | 0.723 | 1.035 |
| RL7A | RPL7A | 60S ribosomal protein L7a | 266 | 0 | 10 |  | 0 | 37 | 2.032E-05 | 0.667 | 3.125 |
| RL18 | RPL18 | 60S ribosomal protein L18 | 188 | 1 | 11 |  | 5 | 52 | 7.498E-03 | 0.651 | 1.280 |
| EF2 | EEF2 | Elongation factor 2 | 858 | 2 | 11 |  | 4 | 40 | 2.257E-02 | 0.630 | 1.163 |
| MIF | MIF | Macrophage migration inhibitory factor | 115 | 2 | 11 |  | 6 | 58 | 7.129E-03 | 0.627 | 1.220 |
| TPM3 | TPM3 | Tropomyosin alpha-3 chain | 284 | 1 | 10 |  | 5 | 102 | 4.128E-07 | 0.626 | 2.237 |
| RL13 | RPL13 | 60S ribosomal protein L13 | 211 | 2 | 11 |  | 11 | 89 | 3.766E-03 | 0.609 | 1.071 |
| S10A8 | S100A8 | Protein S100-A8 | 93 | 0 | 9 |  | 0 | 85 | 8.080E-11 | 0.600 | 4.299 |
| RS16 | RPS16 | 40S ribosomal protein S16 | 146 | 0 | 9 |  | 0 | 27 | 2.880E-04 | 0.600 | 2.687 |
| TMEDA | TMED10 | Transmembrane emp24 domain-containing protein 10 | 219 | 1 | 10 |  | 3 | 35 | 1.925E-02 | 0.598 | 1.281 |
| CAP1 | CAP1 | Adenylyl cyclase-associated protein 1 | 475 | 1 | 10 |  | 4 | 37 | 3.809E-02 | 0.582 | 1.054 |
| ARP2 | ACTR2 | Actin-related protein 2 | 394 | 1 | 9 |  | 2 | 50 | 1.901E-04 | 0.569 | 2.168 |
| VINC | VCL | Vinculin | 1134 | 1 | 9 |  | 3 | 70 | 1.372E-05 | 0.567 | 2.257 |
| PRDX3 | PRDX3 | Thioredoxin-dependent peroxide reductase, mitochondrial | 256 | 1 | 9 |  | 2 | 30 | 1.469E-02 | 0.563 | 1.454 |
| S10A9 | S100A9 | Protein S100-A9 | 114 | 0 | 8 |  | 0 | 109 | 1.732E-13 | 0.533 | 4.654 |
| EZRI | EZR | Ezrin | 586 | 0 | 8 |  | 0 | 69 | 4.951E-09 | 0.533 | 4.003 |
| FRIH | FTH1 | Ferritin heavy chain | 183 | 0 | 8 |  | 0 | 40 | 9.239E-06 | 0.533 | 3.234 |
| RA1L2 | HNRNPA1L2 | Heterogeneous nuclear ribonucleoprotein A1-like 2 | 320 | 1 | 9 |  | 5 | 54 | 5.263E-03 | 0.532 | 1.333 |
| CALD1 | CALD1 | Caldesmon | 793 | 1 | 8 |  | 5 | 52 | 7.498E-03 | 0.530 | 1.280 |
| RS10 | RPS10 | 40S ribosomal protein S10 | 165 | 2 | 9 |  | 4 | 40 | 2.257E-02 | 0.509 | 1.163 |
| PSME1 | PSME1 | Proteasome activator complex subunit 1 | 249 | 2 | 9 |  | 6 | 55 | 1.175E-02 | 0.502 | 1.145 |
| K2C6A | KRT6A | Keratin, type II cytoskeletal 6A | 564 | 0 | 7 |  | 0 | 156 | 1.084E-18 | 0.467 | 5.168 |
| TCP4 | SUB1 | Activated RNA polymerase II transcriptional coactivator p15 | 127 | 0 | 7 |  | 0 | 20 | 1.903E-03 | 0.467 | 2.276 |
| ALDH2 | ALDH2 | Aldehyde dehydrogenase, mitochondrial | 517 | 2 | 8 |  | 7 | 65 | 5.627E-03 | 0.443 | 1.195 |
| COPD | ARCN1 | Coatomer subunit delta | 511 | 2 | 8 |  | 4 | 37 | 3.809E-02 | 0.442 | 1.054 |
| PRDX4 | PRDX4 | Peroxiredoxin-4 | 271 | 1 | 7 |  | 4 | 69 | 8.381E-05 | 0.430 | 1.932 |
| AMPL | LAP3 | Cytosol aminopeptidase | 519 | 1 | 7 |  | 2 | 29 | 1.808E-02 | 0.424 | 1.407 |
| HYOU1 | HYOU1 | Hypoxia up-regulated protein 1 | 999 | 1 | 7 |  | 2 | 28 | 2.224E-02 | 0.422 | 1.359 |
| PERP1 | PACAP | Plasma cell-induced resident endoplasmic reticulum protein | 189 | 0 | 6 |  | 0 | 43 | 4.210E-06 | 0.400 | 3.335 |
| GDIR2 | ARHGDIB | Rho GDP-dissociation inhibitor 2 | 201 | 0 | 6 |  | 0 | 23 | 8.435E-04 | 0.400 | 2.467 |
| LMAN1 | LMAN1 | Protein ERGIC-53 | 510 | 0 | 6 |  | 0 | 19 | 2.501E-03 | 0.400 | 2.207 |
| DX39A | DDX39A | ATP-dependent RNA helicase DDX39A | 427 | 0 | 6 |  | 0 | 16 | 5.712E-03 | 0.400 | 1.975 |
| COR1A | CORO1A | Coronin-1A | 461 | 2 | 7 |  | 8 | 87 | 3.615E-04 | 0.394 | 1.444 |
| K1C16 | KRT16 | Keratin, type I cytoskeletal 16 | 473 | 3 | 7 |  | 12 | 119 | 8.245E-05 | 0.369 | 1.372 |
| PERM | MPO | Myeloperoxidase | 745 | 0 | 5 |  | 0 | 65 | 1.390E-08 | 0.333 | 3.918 |
| 1C12 | HLA-C | HLA class I histocompatibility antigen, Cw-12 alpha chain | 366 | 0 | 5 |  | 0 | 46 | 1.922E-06 | 0.333 | 3.430 |
| IDHP | IDH2 | Isocitrate dehydrogenase [NADP], mitochondrial | 452 | 0 | 5 |  | 0 | 28 | 2.205E-04 | 0.333 | 2.737 |
| 6PGD | PGD | 6-phosphogluconate dehydrogenase, decarboxylating | 483 | 0 | 5 |  | 0 | 18 | 3.289E-03 | 0.333 | 2.134 |
| NIBL1 | FAM129B | Niban-like protein 1 | 746 | 0 | 5 |  | 0 | 18 | 3.289E-03 | 0.333 | 2.134 |
| CPNS1 | CAPNS1 | Calpain small subunit 1 | 268 | 0 | 5 |  | 0 | 17 | 4.332E-03 | 0.333 | 2.057 |
| RL4 | RPL4 | 60S ribosomal protein L4 | 427 | 0 | 5 |  | 0 | 16 | 5.712E-03 | 0.333 | 1.975 |
| HNRH1 | HNRNPH1 | Heterogeneous nuclear ribonucleoprotein H | 449 | 0 | 5 |  | 0 | 16 | 5.712E-03 | 0.333 | 1.975 |
| RL11 | RPL11 | 60S ribosomal protein L11 | 178 | 0 | 5 |  | 0 | 14 | 9.978E-03 | 0.333 | 1.797 |
| WDR1 | WDR1 | WD repeat-containing protein 1 | 606 | 0 | 5 |  | 0 | 12 | 1.756E-02 | 0.333 | 1.594 |
| RL27A | RPL27A | 60S ribosomal protein L27a | 148 | 0 | 5 |  | 0 | 12 | 1.756E-02 | 0.333 | 1.594 |
| TENA | TNC | Tenascin | 2201 | 4 | 14 |  | 175 | 283 | 8.037E-15 | 0.271 | -1.132 |
| K2C5 | KRT5 | Keratin, type II cytoskeletal 5 | 590 | 0 | 4 |  | 0 | 93 | 1.039E-11 | 0.267 | 4.427 |
| TKT | TKT | Transketolase | 623 | 0 | 4 |  | 0 | 25 | 4.923E-04 | 0.267 | 2.581 |
| IGHM | IGHM | Ig mu chain C region | 452 | 0 | 4 |  | 0 | 25 | 4.923E-04 | 0.267 | 2.581 |
| 1433B | YWHAB | 14-3-3 protein beta/alpha | 246 | 0 | 4 |  | 0 | 23 | 8.435E-04 | 0.267 | 2.467 |
| LEG3 | LGALS3 | Galectin-3 | 250 | 0 | 4 |  | 0 | 16 | 5.712E-03 | 0.267 | 1.975 |
| SPCS2 | SPCS2 | Signal peptidase complex subunit 2 | 226 | 0 | 4 |  | 0 | 15 | 7.544E-03 | 0.267 | 1.889 |
| MOGS | MOGS | Mannosyl-oligosaccharide glucosidase | 837 | 0 | 4 |  | 0 | 14 | 9.978E-03 | 0.267 | 1.797 |
| CNDP2 | CNDP2 | Cytosolic non-specific dipeptidase | 475 | 0 | 4 |  | 0 | 13 | 1.322E-02 | 0.267 | 1.699 |
| RS13 | RPS13 | 40S ribosomal protein S13 | 151 | 0 | 4 |  | 0 | 12 | 1.756E-02 | 0.267 | 1.594 |
| 2B14 | HLA-DRB1 | HLA class II histocompatibility antigen, DRB1-4 beta chain | 266 | 0 | 4 |  | 0 | 11 | 2.338E-02 | 0.267 | 1.481 |
| SRSF1 | SRSF1 | Serine/arginine-rich splicing factor 1 | 248 | 0 | 4 |  | 0 | 10 | 3.121E-02 | 0.267 | 1.358 |
| MYOF | MYOF | Myoferlin | 2061 | 0 | 4 |  | 0 | 10 | 3.121E-02 | 0.267 | 1.358 |
| NPM | NPM1 | Nucleophosmin | 294 | 0 | 4 |  | 0 | 9 | 4.180E-02 | 0.267 | 1.224 |
| HBB | HBB | Hemoglobin subunit beta | 147 | 5 | 15 |  | 534 | 835 | 1.059E-45 | 0.220 | -1.200 |
| BGH3 | TGFBI | Transforming growth factor-beta-induced protein ig-h3 | 683 | 5 | 15 |  | 80 | 123 | 3.420E-08 | 0.212 | -1.204 |
| TRFL | LTF | Lactotransferrin | 710 | 0 | 3 |  | 0 | 98 | 2.886E-12 | 0.200 | 4.502 |
| K1C14 | KRT14 | Keratin, type I cytoskeletal 14 | 472 | 0 | 3 |  | 0 | 81 | 2.257E-10 | 0.200 | 4.231 |
| ACTN1 | ACTN1 | Alpha-actinin-1 | 892 | 0 | 3 |  | 0 | 47 | 1.481E-06 | 0.200 | 3.460 |
| CATG | CTSG | Cathepsin G | 255 | 0 | 3 |  | 0 | 28 | 2.205E-04 | 0.200 | 2.737 |
| ILEU | SERPINB1 | Leukocyte elastase inhibitor | 379 | 0 | 3 |  | 0 | 15 | 7.544E-03 | 0.200 | 1.889 |
| CD14 | CD14 | Monocyte differentiation antigen CD14 | 375 | 0 | 3 |  | 0 | 13 | 1.322E-02 | 0.200 | 1.699 |
| DCD | DCD | Dermcidin | 110 | 0 | 2 |  | 0 | 13 | 1.322E-02 | 0.200 | 1.699 |
| SET | SET | Protein SET | 290 | 0 | 3 |  | 0 | 12 | 1.756E-02 | 0.200 | 1.594 |
| RCN3 | RCN3 | Reticulocalbin-3 | 328 | 0 | 3 |  | 0 | 12 | 1.756E-02 | 0.200 | 1.594 |
| GSTO1 | GSTO1 | Glutathione S-transferase omega-1 | 241 | 0 | 3 |  | 0 | 11 | 2.338E-02 | 0.200 | 1.481 |
| PGAM1 | PGAM1 | Phosphoglycerate mutase 1 | 254 | 0 | 3 |  | 0 | 10 | 3.121E-02 | 0.200 | 1.358 |
| SC11C | SEC11C | Signal peptidase complex catalytic subunit SEC11C | 192 | 0 | 3 |  | 0 | 10 | 3.121E-02 | 0.200 | 1.358 |
| HNRPQ | SYNCRIP | Heterogeneous nuclear ribonucleoprotein Q | 623 | 0 | 3 |  | 0 | 10 | 3.121E-02 | 0.200 | 1.358 |
| CO3A1 | COL3A1 | Collagen alpha-1(III) chain | 1466 | 5 | 13 |  | 52 | 92 | 1.491E-04 | 0.193 | -1.006 |
| HBA | HBA1 | Hemoglobin subunit alpha | 142 | 5 | 13 |  | 147 | 249 | 1.476E-11 | 0.174 | -1.065 |
| LMNB2 | LMNB2 | Lamin-B2 | 600 | 3 | 9 |  | 40 | 68 | 4.871E-04 | 0.156 | -1.067 |
| LYSC | LYZ | Lysozyme C | 148 | 0 | 2 |  | 0 | 22 | 1.105E-03 | 0.133 | 2.406 |
| STOM | STOM | Erythrocyte band 7 integral membrane protein | 288 | 0 | 2 |  | 0 | 22 | 1.105E-03 | 0.133 | 2.406 |
| H15 | HIST1H1B | Histone H1.5 | 226 | 0 | 2 |  | 0 | 18 | 3.289E-03 | 0.133 | 2.134 |
| PLMN | PLG | Plasminogen | 810 | 0 | 2 |  | 0 | 16 | 5.712E-03 | 0.133 | 1.975 |
| MMP3 | MMP3 | Stromelysin-1 | 477 | 0 | 2 |  | 0 | 15 | 7.544E-03 | 0.133 | 1.889 |
| CATA | CAT | Catalase | 527 | 0 | 2 |  | 0 | 13 | 1.322E-02 | 0.133 | 1.699 |
| PARP1 | PARP1 | Poly [ADP-ribose] polymerase 1 | 1014 | 0 | 2 |  | 0 | 12 | 1.756E-02 | 0.133 | 1.594 |
| RS26 | RPS26 | 40S ribosomal protein S26 | 115 | 0 | 2 |  | 0 | 10 | 3.121E-02 | 0.133 | 1.358 |
| TRFE | TF | Serotransferrin | 698 | 4 | 12 |  | 59 | 81 | 1.433E-07 | 0.126 | -1.367 |
| PRDX2 | PRDX2 | Peroxiredoxin-2 | 198 | 5 | 12 |  | 41 | 67 | 2.206E-04 | 0.117 | -1.122 |
| ACADV | ACADVL | Very long-chain specific acyl-CoA dehydrogenase, mitochondrial | 655 | 3 | 9 |  | 36 | 49 | 3.622E-05 | 0.092 | -1.382 |
| PRELP | PRELP | Prolargin | 382 | 4 | 10 |  | 52 | 80 | 9.049E-06 | 0.089 | -1.205 |
| HNRPU | HNRNPU | Heterogeneous nuclear ribonucleoprotein U | 825 | 2 | 7 |  | 16 | 20 | 3.383E-03 | 0.081 | -1.512 |
| LUM | LUM | Lumican | 338 | 4 | 10 |  | 47 | 69 | 1.008E-05 | 0.072 | -1.273 |
| DPYL3 | DPYSL3 | Dihydropyrimidinase-related protein 3 | 570 | 3 | 8 |  | 28 | 40 | 4.910E-04 | 0.067 | -1.318 |
| K2C6C | KRT6C | Keratin, type II cytoskeletal 6C | 564 | 0 | 1 |  | 0 | 64 | 1.799E-08 | 0.067 | 3.896 |
| ANXA3 | ANXA3 | Annexin A3 | 323 | 0 | 1 |  | 0 | 23 | 8.435E-04 | 0.067 | 2.467 |
| K1C17 | KRT17 | Keratin, type I cytoskeletal 17 | 432 | 0 | 1 |  | 0 | 21 | 1.450E-03 | 0.067 | 2.343 |
| ITAM | ITGAM | Integrin alpha-M | 1152 | 0 | 1 |  | 0 | 18 | 3.289E-03 | 0.067 | 2.134 |
| CAMP | CAMP | Cathelicidin antimicrobial peptide | 170 | 0 | 1 |  | 0 | 14 | 9.978E-03 | 0.067 | 1.797 |
| KRT83 | KRT83 | Keratin, type II cuticular Hb3 | 493 | 0 | 1 |  | 0 | 14 | 9.978E-03 | 0.067 | 1.797 |
| IGLL5 | IGLL5 | Immunoglobulin lambda-like polypeptide 5 | 214 | 0 | 1 |  | 0 | 10 | 3.121E-02 | 0.067 | 1.358 |
| ESYT1 | ESYT1 | Extended synaptotagmin-1 | 1104 | 0 | 1 |  | 0 | 9 | 4.180E-02 | 0.067 | 1.224 |
| MMP9 | MMP9 | Matrix metalloproteinase-9 | 707 | 0 | 1 |  | 0 | 9 | 4.180E-02 | 0.067 | 1.224 |
| LDHB | LDHB | L-lactate dehydrogenase B chain | 334 | 2 | 5 |  | 18 | 31 | 2.188E-02 | 0.064 | -1.068 |
| APOA1 | APOA1 | Apolipoprotein A-I | 267 | 3 | 7 |  | 28 | 46 | 2.455E-03 | 0.063 | -1.122 |
| ANX11 | ANXA11 | Annexin A11 | 505 | 2 | 5 |  | 10 | 15 | 4.896E-02 | 0.040 | -1.282 |
| PRG4 | PRG4 | Proteoglycan 4 | 1404 | 3 | 6 |  | 32 | 53 | 1.323E-03 | 0.024 | -1.107 |
| PGS1 | BGN | Biglycan | 368 | 3 | 7 |  | 25 | 29 | 1.024E-04 | -0.027 | -1.609 |
| TPD54 | TPD52L2 | Tumor protein D54 | 206 | 1 | 2 |  | 9 | 9 | 1.145E-02 | -0.033 | -1.813 |
| POSTN | POSTN | Periostin | 836 | 2 | 5 |  | 17 | 13 | 5.517E-05 | -0.082 | -2.170 |
| MIME | OGN | Mimecan | 298 | 1 | 1 |  | 6 | 4 | 1.326E-02 | -0.093 | -2.278 |
| DYHC1 | DYNC1H1 | Cytoplasmic dynein 1 heavy chain 1 | 4646 | 1 | 1 |  | 4 | 2 | 2.878E-02 | -0.111 | -2.504 |
| PHB | PHB | Prohibitin | 272 | 3 | 5 |  | 16 | 17 | 1.037E-03 | -0.119 | -1.732 |
| CO3 | C3 | Complement C3 | 1663 | 4 | 8 |  | 41 | 41 | 3.965E-08 | -0.133 | -1.815 |
| CO5A2 | COL5A2 | Collagen alpha-2(V) chain | 1499 | 1 | 1 |  | 7 | 2 | 6.821E-04 | -0.141 | -3.157 |
| S10AA | S100A10 | Protein S100-A10 | 97 | 2 | 2 |  | 5 | 4 | 3.762E-02 | -0.163 | -2.064 |
| EIF3C | EIF3C | Eukaryotic translation initiation factor 3 subunit C | 913 | 2 | 2 |  | 6 | 4 | 1.326E-02 | -0.187 | -2.278 |
| A1AG1 | ORM1 | Alpha-1-acid glycoprotein 1 | 201 | 1 | 0 |  | 2 | 0 | 3.799E-02 | -0.200 | -3.191 |
| ATPO | ATP5O | ATP synthase subunit O, mitochondrial | 213 | 1 | 0 |  | 2 | 0 | 3.799E-02 | -0.200 | -3.191 |
| COR1B | CORO1B | Coronin-1B | 489 | 1 | 0 |  | 2 | 0 | 3.799E-02 | -0.200 | -3.191 |
| RL27 | RPL27 | 60S ribosomal protein L27 | 136 | 1 | 0 |  | 2 | 0 | 3.799E-02 | -0.200 | -3.191 |
| ERP44 | ERP44 | Endoplasmic reticulum resident protein 44 | 406 | 1 | 0 |  | 2 | 0 | 3.799E-02 | -0.200 | -3.191 |
| SNX3 | SNX3 | Sorting nexin-3 | 162 | 1 | 0 |  | 2 | 0 | 3.799E-02 | -0.200 | -3.191 |
| SAHH | AHCY | Adenosylhomocysteinase | 432 | 1 | 0 |  | 2 | 0 | 3.799E-02 | -0.200 | -3.191 |
| RAB2A | RAB2A | Ras-related protein Rab-2A | 212 | 1 | 0 |  | 2 | 0 | 3.799E-02 | -0.200 | -3.191 |
| AP2M1 | AP2M1 | AP-2 complex subunit mu | 435 | 1 | 0 |  | 2 | 0 | 3.799E-02 | -0.200 | -3.191 |
| CRAC1 | CRTAC1 | Cartilage acidic protein 1 | 661 | 1 | 0 |  | 2 | 0 | 3.799E-02 | -0.200 | -3.191 |
| S10AD | S100A13 | Protein S100-A13 | 98 | 1 | 0 |  | 2 | 0 | 3.799E-02 | -0.200 | -3.191 |
| IMMT | IMMT | Mitochondrial inner membrane protein | 758 | 1 | 0 |  | 2 | 0 | 3.799E-02 | -0.200 | -3.191 |
| CBPA3 | CPA3 | Mast cell carboxypeptidase A | 417 | 1 | 0 |  | 2 | 0 | 3.799E-02 | -0.200 | -3.191 |
| BDH2 | BDH2 | 3-hydroxybutyrate dehydrogenase type 2 | 245 | 1 | 0 |  | 2 | 0 | 3.799E-02 | -0.200 | -3.191 |
| UBE2N | UBE2N | Ubiquitin-conjugating enzyme E2 N | 152 | 1 | 0 |  | 2 | 0 | 3.799E-02 | -0.200 | -3.191 |
| TGM2 | TGM2 | Protein-glutamine gamma-glutamyltransferase 2 | 687 | 1 | 0 |  | 2 | 0 | 3.799E-02 | -0.200 | -3.191 |
| CILP1 | CILP | Cartilage intermediate layer protein 1 | 1184 | 1 | 0 |  | 2 | 0 | 3.799E-02 | -0.200 | -3.191 |
| PEDF | SERPINF1 | Pigment epithelium-derived factor | 418 | 1 | 0 |  | 2 | 0 | 3.799E-02 | -0.200 | -3.191 |
| CO5A1 | COL5A1 | Collagen alpha-1(V) chain | 1838 | 1 | 0 |  | 2 | 0 | 3.799E-02 | -0.200 | -3.191 |
| ELAV1 | ELAVL1 | ELAV-like protein 1 | 326 | 1 | 0 |  | 2 | 0 | 3.799E-02 | -0.200 | -3.191 |
| CILP2 | CILP2 | Cartilage intermediate layer protein 2 | 1156 | 1 | 0 |  | 2 | 0 | 3.799E-02 | -0.200 | -3.191 |
| OAS3 | OAS3 | 2'-5'-oligoadenylate synthase 3 | 1087 | 1 | 0 |  | 2 | 0 | 3.799E-02 | -0.200 | -3.191 |
| CO5A3 | COL5A3 | Collagen alpha-3(V) chain | 1745 | 1 | 0 |  | 2 | 0 | 3.799E-02 | -0.200 | -3.191 |
| RAB14 | RAB14 | Ras-related protein Rab-14 | 215 | 1 | 0 |  | 3 | 0 | 7.547E-03 | -0.200 | -3.578 |
| PGS2 | DCN | Decorin | 359 | 1 | 0 |  | 4 | 0 | 1.527E-03 | -0.200 | -3.883 |
| AL1A1 | ALDH1A1 | Retinal dehydrogenase 1 | 501 | 1 | 0 |  | 4 | 0 | 1.527E-03 | -0.200 | -3.883 |
| HP1B3 | HP1BP3 | Heterochromatin protein 1-binding protein 3 | 553 | 1 | 0 |  | 5 | 0 | 3.131E-04 | -0.200 | -4.134 |
| COL12 | COLEC12 | Collectin-12 | 742 | 1 | 0 |  | 5 | 0 | 3.131E-04 | -0.200 | -4.134 |
| CBPQ |  | Plasma glutamate carboxypeptidase | 472 | 1 | 0 |  | 6 | 0 | 6.486E-05 | -0.200 | -4.349 |
| AEBP1 | AEBP1 | Adipocyte enhancer-binding protein 1 | 1158 | 1 | 0 |  | 6 | 0 | 6.486E-05 | -0.200 | -4.349 |
| COFA1 | COL15A1 | Collagen alpha-1(XV) chain | 1388 | 1 | 0 |  | 7 | 0 | 1.354E-05 | -0.200 | -4.535 |
| MYH11 | MYH11 | Myosin-11 | 1972 | 1 | 0 |  | 10 | 0 | 1.273E-07 | -0.200 | -4.983 |
| HBG2 | HBG2 | Hemoglobin subunit gamma-2 | 147 | 1 | 0 |  | 25 | 0 | 1.278E-17 | -0.200 | -6.207 |
| ASPN | ASPN | Asporin | 380 | 2 | 2 |  | 24 | 12 | 1.693E-08 | -0.222 | -2.744 |
| COX2 | MT-CO2 | Cytochrome c oxidase subunit 2 | 227 | 2 | 1 |  | 4 | 2 | 2.878E-02 | -0.244 | -2.504 |
| SAR1A | SAR1A | GTP-binding protein SAR1a | 198 | 2 | 1 |  | 4 | 2 | 2.878E-02 | -0.244 | -2.504 |
| APOE | APOE | Apolipoprotein E | 317 | 1 | 1 |  | 9 | 4 | 4.480E-04 | -0.256 | -2.778 |
| TENX | TNXB | Tenascin-X | 4289 | 3 | 5 |  | 76 | 68 | 1.430E-15 | -0.265 | -1.976 |
| HBD | HBD | Hemoglobin subunit delta | 147 | 4 | 5 |  | 245 | 216 | 9.342E-48 | -0.269 | -2.012 |
| CO4A | C4A | Complement C4-A | 1744 | 2 | 1 |  | 13 | 4 | 3.329E-06 | -0.290 | -3.254 |
| CAH1 | CA1 | Carbonic anhydrase 1 | 261 | 4 | 4 |  | 34 | 31 | 1.411E-07 | -0.291 | -1.943 |
| COCA1 | COL12A1 | Collagen alpha-1(XII) chain | 3063 | 2 | 1 |  | 17 | 5 | 6.862E-08 | -0.294 | -3.359 |
| COEA1 | COL14A1 | Collagen alpha-1(XIV) chain | 1796 | 4 | 6 |  | 167 | 108 | 4.410E-43 | -0.329 | -2.449 |
| FMOD | FMOD | Fibromodulin | 376 | 2 | 0 |  | 8 | 0 | 2.845E-06 | -0.400 | -4.700 |
|  |  |  |  |  |  |  |  |  |  |  |  |

1. Proteins are listed in descending order of *SpI*-value, and “_HUMAN” are removed from UniProtKG entry names.

Supplemental Figure 1. Example of TIC chromatographic profiles. Triplicate runs for samples OA01 and RA01.

Supplemental Figure 2. The fold changes of three of the representative proteins (S100A8, RS9 and PERP1) in log2 comparing the peak areas extracted from LC-MS raw data with the spectral counts. Therein, [RA]/[OA] denotes the ratio of peak area between RA and OA. The peak areas of SHEESHKE (doubly charged), ELSELVYTDVLDR (doubly charged), and ELAPYDENWFYTR (doubly charged), were used to calculate the log_2_ ([RA]/[OA]) for S100A8, PERP1, and RS19 respectively. For spectral counts, we used *R_SC_* –values (spectral counting-based fold-change in log2) given by Old MW et al (Mol Cell Proteomics 2005, 4:1487–502.).
